# Dietary intake of Aboriginal Australian children aged 6–36 months in a remote community: a cross-sectional study

**DOI:** 10.1186/s12937-020-00550-y

**Published:** 2020-04-15

**Authors:** Emma Tonkin, Dani Kennedy, Sarah Hanieh, Beverley-Ann Biggs, Therese Kearns, Veronica Gondarra, Roslyn Dhurrkay, Julie Brimblecombe

**Affiliations:** 1 Nutrition, Dietetics and Food, Faculty of Medicine, Nursing and Health Sciences, Monash University, Notting Hill, Victoria 3168 Australia; 2 College of Medicine and Public Health, Flinders University, Adelaide, South Australia 5042 Australia; 3 Nutrition Program, Wellbeing and Preventable Chronic Disease, Menzies School of Health Research, Casuarina, Northern Territory 0810 Australia; 4 Department of Medicine at the Peter Doherty Institute for Infection and Immunity, The University of Melbourne, Melbourne, Victoria 3010 Australia; 5 Child Health, Menzies School of Health Research, Spring Hill, Queensland 4000 Australia

**Keywords:** Indigenous population, Child health, Food intake, Food security

## Abstract

**Background:**

Scarce literature comprehensively captures the transition to solid foods for children in remote Aboriginal Australian communities, a population expected to be especially vulnerable to nutritional inadequacy for largely socio-economic reasons. This study describes the dietary intake of children aged 6–36 months in a remote Aboriginal community during the years of solids introduction and establishment. Specifically, we aimed to explore milk feeding practices, major sources of nutrition and traditional food consumption, dietary patterns and nutrient and food group intakes, and compare these to national and international recommendations.

**Methods:**

This dietary assessment was conducted as part of an observational, cross-sectional Child Health and Nutrition study. Three 24-h dietary recalls were completed with the parent/care-giver of each participant over 2–4 weeks, capturing a pay-week, non-pay-week and weekend day from October 2017–February 2018. Additional information collected included sociodemographic data, food security status, usual cooking practices, and attendance at playgroup.

**Results:**

Diet histories for 40 children were included in the analysis (~ 40% of the population). Breast feeding rates were high (85%), with mothers exclusively feeding on demand. Very few participants met recommended intakes for wholegrains (*n* = 4, 10%), vegetables (*n* = 7, 18%), dairy (*n* = 5, 18%) and fruit (*n* = 13, 33%), while more children met the guidelines for meat (*n* = 19, 48%) and discretionary food intake (*n* = 28, 70%). Traditional foods were always nutritionally dense and consumed frequently (*n* = 22, 55% of children). Statistically significant pay-cycle differences in intakes of all macro-, and numerous micro-nutrients were observed.

**Conclusions:**

Many positive early feeding practices are currently enacted in remote Aboriginal communities including responsive and long duration breastfeeding, and nutrient-dense traditional food consumption from earliest solids introduction. However, the non-pay-week/pay-week cycle is impacting the quality and quantity of children’s diets at a time of rapid growth and development.

## Background

The first 1000 days of life, from conception to 2 years of age, is increasingly acknowledged as critical in setting the course for lifelong health [1] and preventing chronic diseases in later life [2]. This period includes the years of solid food introduction and establishment, so it is essential children receive a nutritionally adequate and diverse diet during this time to ensure optimal growth and development [3, 4], and appropriate taste preferences [5, 6]. Despite this, no data are collected for children under 2 years of age in Australian national nutrition surveys [7, 8]. A number of recent dietary studies in this age group in Australia have reported that diets are lacking in fruit and vegetables [9, 10] and iron [11, 12], and are high in discretionary foods [9, 10, 13]. However, very little literature comprehensively captures the transition to solid foods for children in remote Aboriginal communities, a population expected to be especially vulnerable to nutritional inadequacy.

People living in remote Aboriginal communities face many food system challenges which make them at high risk to food insecurity and nutritional inadequacy. Food insecurity is defined as the lack of physical and economic access to sufficient, safe and nutritious foods that meet the dietary needs and food preferences for an active and healthy life [14]. Australian Aboriginal Peoples for millennia lived in balance with their environments and secured adequate nutritious food through an intimate knowledge of the natural food system. Settler colonisation of Australia drastically altered and/or in many places destroyed this balance and resulted in an over 200-year struggle of Aboriginal Peoples for their rights as first Australians. Seasonal disrupted access to communities for food deliveries [10], high relative food costs (on average a 60–68% increase in cost compared to local centres) [15], and poor access to food storage and preparation equipment [16] as a consequence of socio-economic inequities, increase the risk of food insecurity for people living remotely. Consequently, Brimblecombe, Maypilama [16] found adults living in remote communities reported food insecurity issues impacting food choices and therefore diet quality; for example, choosing high carbohydrate staples that provide calories at cheaper cost and last longer than fresh foods that are not as filling and will rapidly deteriorate. This study also documented the impact of ‘mayla week’, or non-pay week, where there was little money available for food [16]. In Australia it is common to have income from an employer paid in fortnightly instalments, resulting in a week when income is deposited into a bank account (‘pay week’), and a week when it is not (non-pay week). Social welfare is also provided by default in fortnightly instalments resulting in the same pay cycle. Researchers utilising store sales data from remote communities have since shown that community-level dietary quality is significantly reduced during the non-pay week [17], and the pay cycle has also been found to impact dietary diversity in children less than 2 years of age [10].

International studies exploring the coping strategies used by food insecure households have shown families increase their consumption of traditional foods (defined here as wild caught/hunted or harvested plants and animals) and share food between extended family [18]. Given the nutritional density of typical traditional foods this is a valuable strategy, and it is not surprising then that research has found increased dietary quality in association with increased access to traditional foods among children in childcare centres in remote Indigenous communities in Canada [19]. Consumption of traditional foods in general is not captured using store sales data and therefore, whether traditional foods can fully compensate for poor access to food markets in these complex social settings remains unexplored.

Food insecurity has been linked with poorer anthropometric outcomes and chronic energy deficiency in children from birth to 5 years [18, 20, 21], and iron deficiency anaemia in infants and toddlers [22]. This makes a comprehensive comparison of child diets with recommendations in vulnerable populations essential, however no study has previously conducted such an examination of children in remote Aboriginal communities.

The aim of this study was to describe the dietary intake of children aged 6–36 months in a remote Aboriginal community during the years of solids introduction and establishment. Specifically, we aimed to explore milk feeding practices, major sources of nutrition and traditional food consumption, dietary patterns and nutrient and food group intakes to compare with recommendations from the Australian Dietary Guidelines (ADGs).

## Methods

### Study design

A cross-sectional study was conducted to comprehensively document the health and nutrition status of Aboriginal children aged 0–24 months living in a remote Aboriginal community in the Top End of the Northern Territory, Australia (Child Health and Nutrition Study) between July 24th and October 30th 2017. The study reported here is a sub-study describing the dietary intake of enrolled participants.

### Ethics and consent

Written consent from the legal guardian of the child was obtained for all participants. Consultation with Indigenous leaders and community members was undertaken and informed study design, and approval was granted from the Local Council Authority.

### Research team

Local Aboriginal Health Practitioners and community-based researchers were employed and attended a 5-day face to face training program prior to the commencement of the study. The training was conducted by the Investigators and experienced senior Aboriginal Health Practitioners and community-based researchers. Survey questions and protocols were modified during this time to ensure they were appropriate to purpose and the local context. The trained researchers were then mentored ‘on-the-job’ in the community until confident in the dietary intake assessment procedure.

### Participants and sampling

All children living in the community and surrounding Homelands aged 0–24 months at recruitment were eligible to participate in the initial Child Health and Nutrition study. Based on the Primary Health Care service’s records, the total population of children meeting these criteria at the time was approximately 100. Eligible children were identified through staff at the local Primary Health Care services, playgroup and opportunistically within the community. Aboriginal Health Practitioners and Aboriginal community-based researchers were trained in study procedures and explained the research project to caregivers and obtained written informed consent from those choosing to participate. Children were eligible to participate in this dietary sub-study if complete health status data was available for the child and informed consent was obtained from the carer. Dietary data collection was undertaken from October 2017 to February 2018, after recruitment to the Child Health and Nutrition study, therefore, enrolled children were aged between 6 and 36 months at the time of dietary assessment.

### Data collection

#### Socio-demographic

Maternal and infant sociodemographic factors were assessed using a standardized questionnaire administered by the research staff at enrolment using Research Electronic Data Capture (RedCap). The questionnaire included information on demographics, maternal occupation, education, environmental factors, smoking history, playgroup attendance, hygiene factors and food security. Information was asked in local language and recorded in English.

#### Dietary assessment

Dietary assessment was carried out over three 2-week data collection field trips to the community. Field trips occurred in October and November 2017, and January 2018, therefore during the build-up and tropical monsoon seasons in the area. The dietary data collection team primarily comprised three local Aboriginal researchers (RD, VG, YD) and a nutrition research assistant (DK), supported by two research dietitians (ET and JB). The dietary assessment team was provided with the contact details of participants who had agreed to the dietary assessment component of the growth and nutrition study, and who had a complete set of health measurements. Participants were contacted to confirm informed consent upon further explanation of the dietary data collection process, and to organise a time to meet at participants’ homes, the playgroup or an alternative location of their choosing. Sessions were completed in a combination of English and local language, with the Aboriginal researchers translating as needed.

Three 24-h recalls were completed with each participant over 2–4 weeks. This was to ensure the three recalls captured a pay week, non-pay week and weekend day [10]. Dietary recalls were conducted by either the research assistant (DK) or senior research dietitian (JB), until the three trained local researchers (VG, RD, YD) felt confident to conduct them supervised by the research assistant/dietitian. The 24-h recall procedure followed a standardised three-pass method [23, 24]. The child’s primary carer was asked to detail everything the child ate and drank in the previous 24 h, starting from midnight on the previous day, including dietary supplements (vitamin or mineral supplementation). Food volumes and portion sizes were estimated using household measures (metric cups and spoons). Breastfeeding mothers were asked how many times and for what length of time in minutes they had breastfed for during the previous 24 h. Additional information regarding usual cooking practices was collected using the Menzies Remote Short-Item Dietary Assessment Tool [25]. Information was also collected regarding playgroup attendance as meals are provided at playgroup. After completing the three-pass recall, carers were asked if anyone else had cared for the child during the relevant 24-h period, including other children. These people were followed up where possible and asked if they had provided the child any food or drink, with available details recorded. Dietary intakes were recorded by hand on a hardcopy document, and data were entered into a custom made Access database (Microsoft, USA) and linked withthe 2011–13 AUSNUT food composition tables [26], the community store food product description list, and the Australian Health Survey discretionary food list and food and supplement classification data cubes [27]. Discretionary products are foods and beverages high in added sugar, fat and/or salt [27]. All data were entered by one researcher (DK), and fully cross-checked by a second (ET).

### Analysis

Descriptive statistics were calculated for participant characteristics, food item intakes, macro- and micronutrients and food group intakes. Intakes of nutrients and food groups are reported as averages of the three 24-h recalls. Food group intakes for all participants were compared with the ADGs for Children and Adolescents and Infants and Toddlers [28]. Recommended intakes for children aged < 12 months are presented with smaller serve sizes in the ADGs (for example, a serve of fruit is 20 g) so these were converted to the standard serve sizes (for example, a serve of fruit being 150 g) to ensure they were comparable with the custom database using the Australian Health Survey data cubes. For children 1–3-years-old macro- and micronutrient intakes were compared with recommended intakes from the Nutrient Reference Values (NRVs) for Australia and New Zealand [29]. For children < 12 months of age, for all nutrients excepting zinc and iron, the NRVs estimate adequate intake of nutrients based on the nutrient content of human milk, therefore it would not be meaningful to compare these children’s solids intake against this standard. As such, we have reported these intakes but not compared them to any standard.

Exploratory inferential statistics were also conducted. Paired sample t-tests and Wilcoxon signed rank tests were used to assess the impact of the pay cycle on dietary intakes. Average intakes for each pay and non-pay week were calculated, and variables log transformed where possible. Given the sample size available for this analysis (*n* = 32), and with two-sided significance set to 0.05 and 80% power, the minimally detectable effect was a mean difference in energy of 690 kJ (SD 1350 kJ) between pay and non-pay weeks. Two-sided Fisher’s exact tests were used to assess any difference in prevalence of children meeting recommended intakes based on traditional food consumption, attendance at playgroup/formal care and food security status. Dietary intakes of food groups and key nutrients were also compared between children eating traditional food and attending playgroup with T-tests and Mann-Whitney U tests. IBM SPSS Statistics version 25 (IBM Corp, Armonk, NY, USA) was used for statistical analyses.

## Results

**Fig. 1:**
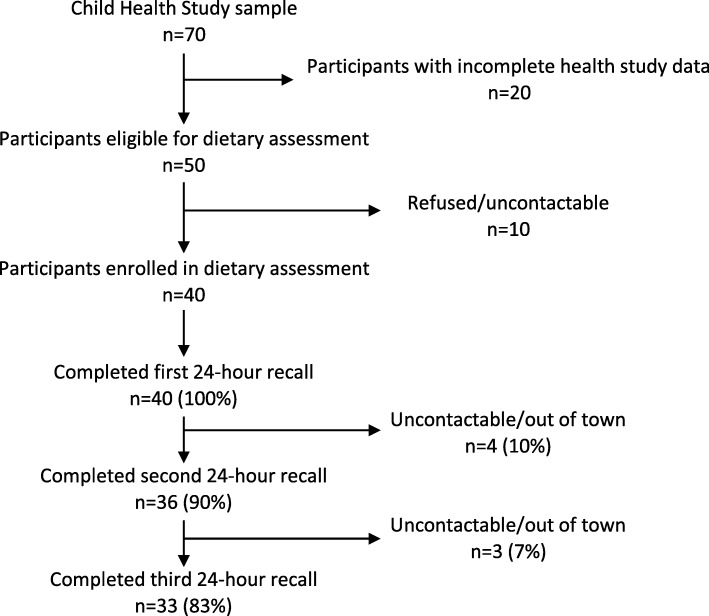
Flow diagram of participants’ involvement with the dietary assessment component of the Child Health Study

Forty of the 70 participants of the Child Health and Nutrition study participated in dietary assessment (Fig. 1). The time of follow up ranged from 6 to 30 days, with a median of 16 days between 1st and 3rd recalls. Carers reported their child ate less than usual at 21 (20%) of the 24-h recall sessions, more than usual at 22 (20%), and their usual intake at 65 (60%) sessions. Carers explained that children ate less than usual due to no money (*n* = 8, 38%) or illness (*n* = 7, 33%), card payment facilities being down at the store (*n* = 2, 10%), the child refused to eat (*n* = 2, 10%), the family was out hunting (*n* = 1, 5%) and the child was simply not hungry (*n* = 1, 5%). All carers reported that their child ate more than usual because they were very hungry (*n* = 21, 96%), except one who explained this was due to reduced milk feedings. Two children received an iron injection (5%), two received oral Vitamin C tablets (5%), one received oral iron supplementation (3%, 3 doses over 3 days), and one received a variety of oral supplements including a multivitamin, folic acid and zinc (3%) (Table 1).

**Table 1:** Participant characteristics

	***n*** (%)
Gender	
Female	20 (50)
Male	20 (50)
Age at first session	
6–9 months	5 (13)
9–12 months	7 (18)
1–2 years	22 (55)
> 2 years	6 (15)
Use of dietary supplements	
Yes	6 (15)
No	34 (85)
Currently breastfeeding	
< 12 months	11 (92)
1–2 years	19 (86)
> 2 years	4 (67)
Reported to attend formal care/playgroup	32 (80)

### Milk feeding

A high proportion (*n* = 34, 85%) of children were breast fed, including those > 2 years old (*n* = 4, 67%). Of the six children who were not breastfed, two received formula (12 and 20 months old), and four did not (10, 19, 27 and 34 months old). The only other child to receive formula was also breastfed. This child was a twin and given ‘top-up’ formula feeds. All three children were provided a Newborn/Stage 1 formula as this was the only type available in the local store. Carers reported they had introduced solids between four and 6 months of age.

Mothers exclusively reported breastfeeding on-demand and therefore found it extremely difficult to provide detail regarding frequency and duration of milk feeds. Mothers described co-sleeping with their children with children latching to feed throughout the night, often without the mother’s full awareness. As such, quantifying breast milk intake was not possible. The most specific data able to be collected were an approximate number of feeds, and whether these were short or long in duration (this was suggested to participants as </> 10 min). Feeding patterns followed a progression of more frequent feeds in younger age groups (average of 4 long and 2 short feeds, 6–11 months), tapering off in the older age groups (average of 2 long and 1 short feed, 12–18 months; average of 1 long and 1 short, 18+ months). Mothers reported breastfeeding more to supplement solids when food was scarce and encouraging more breastfeeding when children were unwell.

### Meal patterns and major sources of nutrition

Breastfeeds were typically provided in the early morning, between meals and before bed, although there was some variability between and within children reflecting responsive feeding practices. Breakfast was the meal provided the most consistently to all children, eaten at 98 of the 105 recalls where food was consumed (93%), compared with lunch (*n* = 85, 81%), dinner (*n* = 63, 60%) and snacks (*n* = 42, 40%). Similarly, breakfast was typically the most substantial meal of the day, with a breakfast food being the primary source of energy for 20 (50%) children. For the majority of the remaining children (*n* = 16, 40%) a substantial lunch at playgroup or a dinner meal was the major source of energy. On 12 (14%) and 11 (17%) occasions respectively, lunch or dinner consisted of an unsubstantial meal, such as a single chicken wing, piece of plain damper or djepi (flour mixed with water, milk or formula and eaten raw or cooked). Most children were reported to consume a snack at any recall (*n* = 27, 68%), and these varied in quality from fresh whole fruit, yoghurt or infant custard, to potato crisps, sweet biscuits and cake.

### Food group intakes compared with recommendations

Table 2 reports individual food group intakes by age group and compared to recommendations. These intakes exclude human milk and formula feeding, and therefore are a representation of dietary solids and alternative drink intakes.

**Table 2:** Caregiver reported food group intake compared with Australian Dietary Guideline recommended intakes for children aged 6–36 months from a remote Aboriginal community in Northern Australia

Age	< 1 year (***n*** = 12)		1–2 years (***n*** = 22)		2–3 years (***n*** = 6)	
Food group	Average of 24-h recalls, median serves per day (IQR)	Recommended intakes from ADGs (serves)	Average of 24-h recalls, median serves per day (IQR)	Recommended intakes from ADGs (serves)	Average of 24-h recalls, median serves per day (IQR)	Recommended intakes from ADGs (serves)
Vegetables	0.51 (0.15, 0.95)	0.5	0.59 (0.06, 1.01)	2–3	0.76 (0.31, 1.77)	2.5
Fruit	0.45 (0.00, 0.95)	0.07	1.00 (0.03, 1.65)	0.5	0.66 (0.00, 1.85)	1
Fruit *excluding* juice	0.00 (0.00, 0.35)	0.07	0.04 (0.00, 0.65)	0.5	0.24 (0.00, 1.03)	1
Grain	1.24 (0.92, 1.6)	2	1.93 (1.65, 2.53)	4	3.74 (1.95, 5.01)	4
Meat	0.43 (0.20, 0.86)	0.5	0.89 (0.46, 1.33)	1	1.75 (0.51, 2.29)	1
Dairy *excluding* milk feeds	0.38 (0.24, 0.78)	N/A	0.54 (0.36, 1.02)	1–1.5	0.76 (0.47, 0.96)	1.5
Discretionary foods^a^	0.03 (0.00, 0.43)	0	0.16 (0.00, 0.58)	0-1^b^	0.26 (0.10, 0.85)	0–1 ^**b**^

^a^ Defined as foods to be consumed ‘sometimes or in small amounts’ as they are not essential for nutrient requirements and some contain too much fat, sugar, and salt

^b^ Although never recommended, this allowance is for additional 600 kJ serves for children who are more active, taller or older within the age group. Ideally these additional serves would be made up from the five food groups or unsaturated spreads and oils, however discretionary items may be included as per the ADGs

Overall, very few participants met recommended intakes for wholegrain (*n* = 4, 10%), vegetables (*n* = 7, 18%), dairy (*n* = 5, 18%) and fruit when juice was not included (*n* = 13, 33%). Almost half met the recommended intakes for meat (*n* = 19, 48%), for fruit when juice was included (*n* = 24, 60%), and most for discretionary food (*n* = 28, 70%). Significantly more infants met the recommended intake of vegetables compared to the older age groups (< 12 months *n* = 6, 50% compared with 1–2 years *n* = 0, 0% and > 2 years *n* = 1, 17%, *p* = 0.001). Fewer infants met the ADGs for discretionary food consumption (i.e., no discretionary food consumption in infants < 12 months) compared to the recommended guideline for older children (please see footnote ^b^ in Table 2 for this recommendation) (< 12 months: *n* = 5, 42%, met the guideline; 1–2 years: *n* = 18, 82%; > 2 years: *n* = 5, 83%, *p* = 0.04).

### Macro- and micronutrient intakes

Table 3 reports macro- and micronutrient intakes by age group and compared to recommendations. Again, these intakes exclude human milk and formula feeding, and therefore are a representation of dietary solids and alternative drink intakes.

**Table 3:** Caregiver reported macro- and micronutrient intake compared with Nutrient Reference Values for children aged 6–36 months from a remote Aboriginal community in Northern Australia, *excluding* intake from milk feeds

	< 12 months (***n*** = 12)	1–3 years (***n*** = 28)		
Nutrient	Average intake from 24-h recalls, mean, SD or median (IQR)	Average intake from 24-h recalls, mean, SD or median (IQR)	EAR^**a**^	RDI^**b**^ or AI^**c**^ (/day)
Energy (kJ) (excluding milk feeds)	1607.49, 830.02	2982.86 (2124.72, 3684.88)	3200 kJ-5600^d^	
Protein (g)	18.93, 10.25	38.21 (25.67, 46.45)	12.00	14.00
Carbohydrate (g)	48.59, 24.25	75.86 (61.41, 113.42)	–	NP
Dietary fats	11.35, 7.04	25.26 (15.44, 34.75)		
Saturated (g)	4.78, 2.8	10.89 (6.84, 13.30)		-
Monounsaturated (g)	4.27 (1.41, 5.77)	9.12 (4.38, 14.08)		-
Polyunsaturated (g)	1.20 (0.63, 2.20)	2.73 (1.81, 3.80)		-
A-linolenic acid (n-3)	0.15 (0.08, 0.22)	0.29 (0.18, 0.40)		-
(g)	0.96 (0.46, 1.83)	2.33 (1.50, 3.25)		5.00
Linoleic acid (n-6) (g)	16.65 (2.66, 33.47)	8.63 (2.39, 31.90)		-
EPA (mg)	13.48 (5.46, 14.69)	19.57 (9.50, 38.39)		-
DPA (mg)	28.47 (15.64, 47.20)	14.15 (3.70, 39.67)		-
DHA (mg)	210.63, 128.22	414.95 (299.88, 722.91)		-
Trans-fatty acids (mg)				
Dietary fibre (g)	5.16, 3.11	8.93 (5.49, 11.75)	–	14.00
Thiamin (mg)	0.51 (0.30, 0.89)	0.95 (0.67, 1.35)	0.40	0.50
Riboflavin (mg)	0.74 (0.37, 0.86)	1.12 (0.80, 1.50)	0.40	0.50
Niacin equiv. (mg)	5.15, 2.48	10.15 (6.68, 15.14)	5.00	6.00
Vitamin B6 (mg)	0.31 (0.13, 0.42)	0.61, 0.36	0.40	0.50
Vitamin B12 (μg)	1.78, 1.14	2.13 (1.45, 3.11)	0.70	0.90
Folate equiv. (μg)	130.34 (61.58, 211.39)	235.87 (168.35, 348.42)	120.00	150.00
Vitamin A (μg)	131.40 (70.09, 279.24)	249.68 (135.66, 389.27)	210.00	300.00
Vitamin C (mg)	19.39 (1.48, 41.09)	47.38 (13.05, 93.19)	25.00	35.00
Calcium (mg)	190.67 (89.84, 267.30)	260.01 (205.44, 377.62)	360.00	500.00
Zinc (mg)	3.23 (1.95, 6.56)^e^	5.21 (4.57, 9.71)	2.50	3.00
Iron (mg)	3.87, 2.54^f^	6.26 (4.67, 8.66)	4.00	9.00
Magnesium (mg)	70.23 (48.45, 90.99)	117.27 (81.12, 158.42)	65.00	80.00
Iodine (μg)	42.83 (25.88, 72.73)	52.54 (34.33, 89.60)	65.00	90.00
Sodium (mg)	290.74 (207.94, 428.01)	585.70 (444.40, 895.70)	–	200–400.00
Potassium (mg)	609.80, 300.20	1015.28 (730.31, 1549.45)	–	2000.00
Selenium (μg)	23.14 (12.98, 30.15)	40.60 (25.53, 44.48)	20.00	25.00
Vitamin E equiv. (mg)	1.57 (0.65, 2.36)	3.87, 2.16	–	5.00

^a^EAR, estimated average requirement. Defined as ‘a daily nutrient level estimated to meet the requirements of half the healthy individuals a particular life stage and gender group’ [29]

^b^RDI, recommended dietary intake. Defined as ‘the average daily dietary intake level that is sufficient to meet the nutrient requirements of nearly all (97–98%) healthy individuals in a particular life stage and gender group [29]

^c^AI, adequate intake. ‘Defined as ‘the average daily nutrient intake level based on observed or experimentally-determined approximations or estimates of nutrient intake by a group (or groups) of apparently healthy people that are assumed to be adequate’ [29]

^d^This is an estimated energy requirement based on light physical activity for the age group 12 months – 3 years

^e^The EAR for infants < 12 months for zinc is 2.5 mg, and the RDI is 3 mg

^f^The EAR for infants < 12 months for iron is 7 mg, and the RDI is 11 mg

These intakes are consistent with the patterns seen in the food group intake analysis. Table 3 shows energy intake was lower than recommended in 1–3-year-olds, although most of these children were still being breastfed therefore potentially overall energy consumption could be within the recommended range. Protein intake and saturated fat consumption were high, reflecting the high intake of non-lean meats particularly seen in children aged > 2 years (Table 2). Fortified breakfast cereals and breads were chosen by carers, and this was reflected in the adequate and high intakes of thiamin, riboflavin, niacin, vitamin B6 and folate. Weetbix (a fortified wheat-based breakfast cereal) was the primary source of dietary fibre and iron. Despite the inclusion of high-quality lean kangaroo meat in many children’s diets, iron intake was low across all age groups with only five participants (13%) meeting the RDI, with low intakes of green leafy vegetables, nuts, seeds, legumes and pulses likely contributing. Similarly, dietary fibre, vitamins E and A, and potassium were low consistent with low intakes of vegetables and quality grains. Low dairy intakes across all age groups were also likely contributing to inadequate potassium intake, as well as suboptimal intakes of iodine and calcium. The inclusion of traditional foods in the diet, particularly seafood including oysters, supported adequate and high intakes of selenium, vitamin B12 and zinc, with 31 (78%) children meeting the RDI.

### Traditional food consumption

More than half the children were reported to have consumed traditional food at one or more recall session (*n* = 22, 55%), with three (8%) children reported to have consumed traditional food at every recall and five (13%) at more than half their recalls. Of the 46 instances of traditional food consumption, seafood dominated with 19 (41%) being fish or turtle, 13 (28%) molluscs, six (13%) crustaceans, six (13%) kangaroo and one each of seagull eggs and cassava. Most frequently these foods were eaten fresh, typically cooked over a fire on the day of collection.

Despite < 12-month-olds who consumed traditional food at any recall having higher intakes of meat and protein these differences did not reach statistical significance (mean difference 0.41 serves meat, *p* = 0.086 and 12.77 g protein, *p* = 0.056 respectively). These differences were also present although not as pronounced in the 1–3-year-olds (median difference 0.33 serves meat, *p* = 0.68, mean difference in protein 8.39 g, *p* = 0.29). There were no differences in intakes of energy, zinc, and iron between traditional food consumption groups.

### Dietary habits and major intake patterns

#### Dietary habits

Only five children (13%) were reported to consume sugar sweetened beverages including cola, lemonade and cordial, one (3%) was reported to consume tea, while 26 (65%) consumed either full strength or watered down 100% fruit juice, and 18 (45%) ate whole fresh fruit. Carers often explained they would add salt and sugar to their own food, however 31 (77%) carers reported not adding sugar and 30 (75%) not adding salt to children’s meals, and 24 (60%) did not regularly add salt during cooking. Despite 32 (80%) carers reporting that they cut the fat off red meat, and 33 (83%) the skin off chicken, the predominant meats consumed were chicken wings or drumsticks with skin on, and untrimmed beef.

#### Playgroup

Fourteen (35%) children were reported to have had lunch at playgroup at one or more recall sessions. Lunch at playgroup was reported by some carers as a means for providing lunch to children when money was insufficient. Playgroup lunch generally consisted of a substantial balanced meal including meat, vegetables, whole grains (for example a casserole/stew with rice) and fresh fruit. Children 1–3-years-old who attended playgroup had significantly higher intakes of whole fruit (not juice) (median difference 0.17 serves, *p* = 0.006), and all ages were more likely to meet ADGs for discretionary foods if they attended playgroup (*n =* 13, 93% compared with *n* = 15, 58%, *p* = 0.03). There were no other differences in intakes of energy, fibre, protein, iron, zinc, vegetables and grains or in the proportion of children meeting ADGs between the groups.

#### Pay cycle

Carers mentioned financial issues relating to the pay cycle affecting diets as a common struggle (*n* = 21, 53% reported food insecurity), and frequently cited this as the reason children had not consumed lunch or dinner at a recall session. This was reflected in the dietary data. Significantly more energy, carbohydrate, protein, total fat, riboflavin, magnesium, vitamin E, zinc, niacin, potassium and selenium were consumed by children during pay week compared with non-pay week (Table 4). Food group intakes also reflected this pay-cycle trend, but differences were not significant. Similarly, children from households reported to be food secure were more likely to meet the ADGs recommended intake of meat (*n* = 13, 69%) compared with children from food insecure households (*n* = 6, 29%, *p* = 0.012). While the other food groups, iron and zinc showed similar trends, these did not reach statistical significance (for example, the number of children meeting requirements for iron from food secure homes was *n* = 4, 21% compared with *n* = 1, 5%, *p* = 0.172). Carers shared strategies they implemented to reduce the impact of food insecurity on their household, including sharing food/money between extended family, putting money aside during pay week for food in non-pay week, requesting their pay to be deposited weekly, and to go out hunting for wild meats (although this was not reflected in more food insecure children consuming a traditional food at any recall, *p* = 0.726).

**Table 4:** Caregiver reported macro- and micronutrient intakes of children aged 6–36 months from a remote Aboriginal community in Northern Australia *excluding* milk feeds, compared by whether it was the week fortnightly pay was received by the primary carer

	Pay week intake, mean, SD or median (IQR) *n* = 32	Non-pay week intake, mean, SD or median (IQR) *n* = 32	Mean/median difference	***p***-value
Energy (kJ)	3047.83, 1456.57	2300.10, 1172.97	**747.74**	***p*** **= 0.005**
**Macronutrients**				
Protein (g)	33.66 (18.01, 47.21)	27.40 (19.93, 39.13)	**6.26**	***p*** **= 0.03**
Carbohydrate (g)	80.87, 32.49	63.50, 35.75	**17.37**	***p*** **= 0.03**
Total fat (g)	21.43 (13.61, 36.34)	16.03 (9.17, 26.04)	**5.40**	***p*** **= 0.01**
Fibre (g)	7.66 (4.76, 12.39)	5.81 (4.13, 9.90)	1.85	*p* = 0.11
**Key micronutrients**				
Iron (mg)	6.17, 3.29	5.27, 3.12	0.90	*p* = 0.18
Calcium (mg)	270.24, 139.90	241.80, 165.57	28.44	*p* = 0.42
Riboflavin (mg)	1.13, 0.54	0.84, 0.51	**0.28**	***p*** **= 0.03**
Magnesium (mg)	116.77, 60.82	91.06, 43.28	**25.71**	***p*** **= 0.03**
Vitamin B12 (μg)	1.85 (1.30, 2.85)	1.73 (0.85, 2.86)	0.12	*p* = 0.31
Vitamin C (mg)	24.81 (5.70, 69.88)	34.35 (1.73, 62.83)	−9.54	*p* = 0.27
Sodium (mg)	536.91 (343.61, 818.53)	441.37 (195.21, 792.86)	95.54	*p* = 0.07
Vitamin B6 (mg)	0.50 (0.29, 0.87)	0.44 (0.22, 0.64)	0.06	*p* = 0.06
Vitamin E (mg)	3.23 (1.86, 5.72)	2.17 (1.06, 4.10)	**1.06**	***p*** **= 0.03**
Folate equivalents (μg)	225.44 (142.07, 345.49)	186.37 (111.48, 266.09)	39.07	*p* = 0.24
Zinc (mg)	5.36 (3.12, 9.80)	3.28 (2.65, 6.53)	**2.08**	***p*** **= 0.03**
Iodine (μg)	52.28 (42.97, 89.83)	50.62 (18.86, 105.95)	1.66	*p* = 0.20
Thaimin (mg)	0.87 (0.36, 1.34)	0.64 (0.49, 1.08)	0.23	*p* = 0.18
Niacin (mg)	9.34 (4.76, 12.98)	6.22 (5.21, 10.66)	**3.12**	***p*** **= 0.02**
Potassium (mg)	1023.31 (567.60, 1504.58)	826.52 (537.64, 1118.24)	**196.79**	***p*** **= 0.03**
Selenium (μg)	37.63 (22.03, 50.76)	32.50 (17.54, 40.89)	**5.13**	***p*** **= 0.03**
Vitamin A (μg)	192.15 (118.43, 358.26)	170.24 (51.16, 331.43)	21.91	*p* = 0.58
**Food Groups**				
Grain	2.06 (1.47, 3.04)	1.68 (1.13, 3.07)	0.38	*p* = 0.26
Veg	0.46 (0.04, 1.22)	0.40 (0.03, 0.96)	0.06	*p* = 0.34
Fruit	0.53 (0.00, 2.02)	0.03 (0.00, 1.00)	0.50	*p* = 0.12
Meat	0.89 (0.44, 1.62)	0.70 (0.32, 1.11)	0.19	*p* = 0.07
Dairy	0.55 (0.28, 0.98)	0.50 (0.24, 0.86)	0.05	*p* = 0.48
Discretionary	0.05 (0.00, 0.77)	0.00 (0.00, 0.28)	0.05	*p* = 0.36

## Discussion

This study of children aged 6–36 months in a remote Aboriginal community found that during the years of solids introduction and establishment breastfeeding rates were high, consumption of nutrient-dense traditional foods was common, but food insecurity was also prevalent and reflected in significant pay cycle trends in diet quantity and quality. It was found that most children’s diets compared unfavourably with the ADGs, with increasing discrepancy between intakes and the ADGs as children aged. This was due to both low intakes and increasing recommended serves that were not attained by older children. Dairy and vegetable intakes did not vary greatly between the age groups, while meat, fruit and grain intake increased with age-group. Similar dietary patterns were reported in the only other study to compare fruit, vegetable and discretionary food intake in Australian children with the ADGs, despite these children being from the general Australian population [9]. Two additional studies report diets of Aboriginal children during solid food introduction. Similar dietary patterns (low fruit and vegetables, low dairy and low dietary diversity) were found by Leonard, Aquino [10], while children in the study by Smithers, Lynch [30] had substantially more dairy and vegetables, and less meat than reported here. Their sample was approximately half ‘regional/remote’, and therefore it is possible these differences reflect differences in food access.

Breastfeeding is a major community strength, with breastfeeding rates at all ages substantially higher than worldwide reported rates [31] and those of the general Australian population (86% receiving any breastmilk in the second year of life compared with 7.4–18.2% in the general Australian population [32]). Community breastfeeding practice was therefore consistent with World Health Organisation recommendations to continue breastfeeding to 2 years or beyond [31] and to increase breastfeeding frequency during illness. However, contrary to the guidelines most children were reported to receive complementary solids prior to 6 months of age. While local published infant feeding guidelines recommend exclusive breastfeeding to ‘around’ 6 months [33], typically information provided by health services in community is to introduce foods around 6 months of age, and not before four, and community practice reflected this. The most appropriate time for introduction of solids could be debated in this community, with a Cochrane review finding that in populations where newborn iron stores may be suboptimal exclusive feeding until 6 months without iron supplementation may compromise haematologic status [34]. Conversely, infants exclusively breastfed to 6 months had lower rates of gastrointestinal and respiratory infection [34], issues also prevalent in remote communities [35, 36]. As such, it can be recommended that health service advice to mothers less ambiguously recommend exclusive breastfeeding to 6 months, with appropriate iron status screening and supplementation for at risk infants.

Another community strength is the frequency with which traditional foods were provided to children, with rates higher than those reported in a recent study involving First Nations children (55% compared with 36%) [19]. The traditional foods provided were nutrient-dense, containing nutrients known to be critical in this age group such as iron and zinc. Unlike the First Nations study [19] and despite data trends, we were not able to demonstrate significant improvements in dietary quality with traditional food consumption. This is most likely due to the small sample size and relatively large number of age group intake categories required for this analysis resulting in the analysis being underpowered. Nonetheless, these findings are useful for hypothesis generation, with the trends demonstrating the worth of further study. Additionally, based on the nutritional quality of the types of foods found to be consumed, the prevalence of food insecurity reported, and the importance of traditional foods for intergenerational cultural knowledge sharing and eating socialisation, this work supports calls for the prioritisation and protection of access to traditional foods for Australian Aboriginal and Torres Strait Islander people [16, 37], as well as other Indigenous populations internationally [38, 39] as a critical component of dietary improvement in these communities.

Concerningly, although not unexpectedly, food insecurity was reported by more than half the carers. Consistent with this, significant differences between children’s intakes during pay and non-pay weeks were found, with several marginalised nutrients being even more so in non-pay weeks (vitamin E and potassium in particular). While less food insecurity was reported in the study by Leonard, Aquino [10], similar pay cycle trends were found. Although we could not confirm reported increases in breastfeeding frequency during times of food scarcity, it is unlikely this would compensate for the lack of food in children > 12 months old as literature suggests daily breast milk production reduces to just over 200 g at 12 months, and further to 115 g by 2 years [40]. These pay cycle trends reflect complex socio-economic practices overlayed with the interconnecting issues of high food costs and low incomes [15, 16]. This is a community recognised problem, with family and community-level management strategies provided by participants such as sharing between families, and wild harvesting and hunting; these are similar to those reported in international literature [18, 41]. While community services that assist carers in managing food insufficiency such as the provision of substantial lunch meals at playgroup should be celebrated and supported, the fundamental food system challenges that are at the heart of the problem must be addressed for real progress to be made in addressing food insecurity in this setting. Primarily this includes addressing high food costs [41, 42] and providing financial support through increasing the flexibility of welfare and salary payments (for example weekly rather than fortnightly).

Limiting our ability to accurately estimate total macro- and micronutrient intakes was the exclusive practice of breastfeeding on-demand. On-demand feeding makes it difficult to estimate breastfeeding frequency and volume without using highly resource intensive and invasive research procedures such as test-weighing and breast volume assessment. Estimating breastfeed volume has been shown to be difficult in highly controlled conditions [43] therefore, with the added complications that exist with data collection in a remote Aboriginal community, and ethical considerations that research be culturally acceptable in this setting, this estimation becomes impracticable. Despite the difficulties this presents for researchers, Iacovou and Sevilla [44] showed that babies fed on-demand had higher IQ scores and better academic outcomes compared to schedule fed babies. Thus, the responsive breastfeeding practices demonstrated in community through exclusive feeding on-demand should be promoted and protected, and research strategies for measuring intake in this setting could be an area for future research if considered by community to be important.

While the generalisability of this study is limited as it includes children from one remote community, the findings are consistent with findings of similar trends in other remote Australian Aboriginal communities [10], and even Canadian First Nations communities [19], giving confidence that the overall patterns seen are likely to be applicable to many remote communities locally and internationally. Additionally, almost half of the eligible population were included in this analysis, therefore it is a reliable assessment of children’s diets in this community. A further strength of this analysis providing confidence in the validity of the data is the assistance of local Aboriginal researchers in data collection and the capacity building within community the research provided; local researchers with experienced public health nutrition researchers were able to elicit the most detail possible from carers, despite language and cultural differences. In remote Aboriginal communities it is particularly important to have skilled local researchers assist with 24-h recalls to ensure accurate interpretation of the dietary data provided by participants in this unique setting [25]. Finally, the previous work of the research team with remote community food systems enabled the use of a comprehensive database with highly specific food item data entry based on exact knowledge of the composition of products available in community.

## Conclusions

We observed that many positive early feeding practices are currently enacted in remote Aboriginal communities; principally responsive and long duration breastfeeding, and nutrient-dense traditional food consumption from earliest solids introduction. Unfortunately, it was also found that food insecurity caused by high food costs and financial difficulties are impacting on the quality and quantity of children’s diets during the crucial time of rapid growth and development.

## Data Availability

The datasets generated and/or analysed during the current study are not publicly available due ethical and cultural constraint but are available from the corresponding author on reasonable request.

## Abbreviations

ADGs: Australian Dietary Guidelines
NRVs: Nutrient Reference Values
